# Polygenic scores for cardiovascular risk factors improve estimation of clinical outcomes in CCB treatment compared to pharmacogenetic variants alone

**DOI:** 10.1038/s41397-024-00333-2

**Published:** 2024-04-17

**Authors:** Deniz Türkmen, Jack Bowden, Jane A. H. Masoli, João Delgado, Chia-Ling Kuo, David Melzer, Luke C. Pilling

**Affiliations:** 1 Epidemiology & Public Health Group, Department of Clinical & Biomedical Science, Faculty of Health & Life Sciences, University of Exeter, Exeter, UK; 2 Exeter Diabetes Group (ExCEED), Department of Clinical & Biomedical Science, Faculty of Health & Life Sciences, University of Exeter, Exeter, UK; 3 Department of Genetics, Novo Nordisk Research Centre Oxford, Innovation Building, Old Road Campus, Roosevelt Drive, Oxford, UK; 4 Department of Healthcare for Older People, Royal Devon University Healthcare NHS Foundation Trust, Barrack Road, Exeter, UK; 5 UConn Center on Aging, University of Connecticut, Farmington, CT USA; 6 Connecticut Convergence Institute for Translation in Regenerative Engineering, University of Connecticut, Storrs, CT USA

**Keywords:** Risk factors, Genetic association study

## Abstract

Pharmacogenetic variants are associated with clinical outcomes during Calcium Channel Blocker (CCB) treatment, yet whether the effects are modified by genetically predicted clinical risk factors is unknown. We analyzed 32,000 UK Biobank participants treated with dihydropiridine CCBs (mean 5.9 years), including 23 pharmacogenetic variants, and calculated polygenic scores for systolic and diastolic blood pressures, body fat mass, and other patient characteristics. Outcomes included treatment discontinuation and heart failure. Pharmacogenetic variant rs10898815-A (*NUMA1*) increased discontinuation rates, highest in those with high polygenic scores for fat mass. The *RYR3* variant rs877087 T-allele alone modestly increased heart failure risks versus non-carriers (HR:1.13, *p* = 0.02); in patients with high polygenic scores for fat mass, lean mass, and lipoprotein A, risks were substantially elevated (HR:1.55, *p* = 4 × 10^−5^). Incorporating polygenic scores for adiposity and lipoprotein A may improve risk estimates of key clinical outcomes in CCB treatment such as treatment discontinuation and heart failure, compared to pharmacogenetic variants alone.

## Introduction

High blood pressure (BP) is a major modifiable factor affecting cardiovascular disease morbidity and mortality. Dihydropiridine calcium channel blockers (dCCB), e.g., amlodipine, are among the most commonly prescribed first-line treatments for hypertension [1, 2]. Yet the factors influencing patient response and adverse events are poorly understood.

Pharmacogenetic variations can affect drug absorption, metabolism, distribution, excretion, or target, thereby altering drug response [3]. We previously showed that alleles in genes *NUMA1, RYR3, CYP3A5*, *ADRA1A* and *APCDD1* increased risks for adverse outcomes such as heart failure, coronary heart disease, and dCCB discontinuation, in 32,000 patients in UK Biobank receiving dCCB prescriptions in the primary care setting [4]. However, effect sizes were modest; for example, the Hazard Ratio for heart failure in *RYR3* rs877087 T-allele carriers was 1.13 (95% CI 1.02–1.25) versus participants with no T alleles.

dCCBs are lipophilic, with high protein-binding capacity, hepatic metabolism and renal excretion, and are therefore modified by individual patient characteristics such as age, weight, adiposity, baseline blood pressure, biological biomarkers (including serum calcium and urinary sodium), renal and hepatic functions, and lipoprotein [5–11]. Yet the links remain inconclusive [12], likely due to differences in risk factors studied, small sample sizes, a focus on selected patient groups not necessarily representative of clinical practice, and biases common to observational study designs, including confounding and reverse causation. However, individuals inherit germline genetic variants at conception in a random manner that are independent of traditional confounders such as diet and lifestyle. Therefore, substituting these risk factors for genetic proxies should act to minimize the aforementioned biases.

Much work is ongoing to integrate pharmacogenetic information to optimizing treatment effectiveness and reduce side-effects [3, 13–17]. Yet there has been limited discussion on whether genetically determined individual patient characteristics (for example, by using polygenic scores) could be useful predictors of drug response [18]. Polygenic scores reflect individuals’ genetic liability for a trait, derived by summing the number of trait-increasing alleles they carry and weighting each variant’s contribution according to its effect size [19]. Polygenic scores are emerging as important tools for personalized medicine [17] with utility in identifying high-risk patients [20].

We aimed to test whether polygenic scores for risk factors reported to predict CCB outcomes (individual patient characteristics modifying CCB treatment mentioned above) are associated with relevant clinical outcomes, in 32,000 UK Biobank community participants prescribed dCCBs in routine clinical care. We also aimed to test whether adding polygenic score risks with pharmacogenetic variants produced stronger combined associations with selected clinical outcomes.

## Methods

### UK Biobank cohort description

503,325 community-based volunteers aged 40–70 years were recruited in UK Biobank (UKB). The North West Multi-Centre Research Ethics Committee approved the collection and use of UKB data (Research Ethics Committee reference 11/NW/0382). Access to UKB was granted under Application Number 14631. Individual assessments were at one of 22 centers in Wales, Scotland, or England in 2006–2010 [21]. Lifestyles and health information, as well as blood samples for biochemical and genetics analyses, were gathered. General Practice (GP) data are available for 230,096 participants (see below).

### General practice (GP) data

UKB included more than 57 million prescriptions for 230,096 (45.7%) participants from the linked GP data available up to 31 May 2016 (England TPP system) and 31 August 2017 (Wales/Scotland EMIS/Vision system). We analyzed the dihydropyridine subset of CCBs together (herein referred to as dCCBs): for details see the previous analysis [4]. In brief, we identified prescribing information for dCCB medications (amlodipine, felodipine, lacidipine, lercanidipine, nimodipine, nisoldipine, nifedipine, nitrendipine and nicardipine) and dates of prescriptions, using drug codes in clinical Read v2, British National Formulary (BNF), or dm+d (Dictionary of Medicines and Devices) format, depending on suppler. The UK National Institute for Health and Care Excellence (NICE) BNF database (https://bnf.nice.org.uk) was our primary source to detect medication and brand names prescribed in the NHS that met our search criteria (Searched in Oct-Dec 2022).

### Primary outcomes

We ascertained cardiovascular events from hospital inpatient records with up to 14 years follow-up after baseline assessment (HES in England up to 30 September 2021: data from Scotland and Wales censored to 31 August 2020 and 28 February 2018, respectively), covering the period up to the date of censoring of primary care prescribing data. Diagnoses of incident heart failure, coronary heart disease (myocardial infarction/angina) and chronic kidney diseases were ascertained using ICD-10 codes (see supplementary information).

Discontinuation was defined as patients having a date of last prescription at least 90 days prior to the censoring date which is either the date of deduction (removal from GP list, where available) or 28 February 2016 where no deduction date existed. Depending on primary care provider, data after 28 February 2016 was often incomplete (See UK Biobank resource 591 [22]).

### Genotype data

**Fig. 1:**
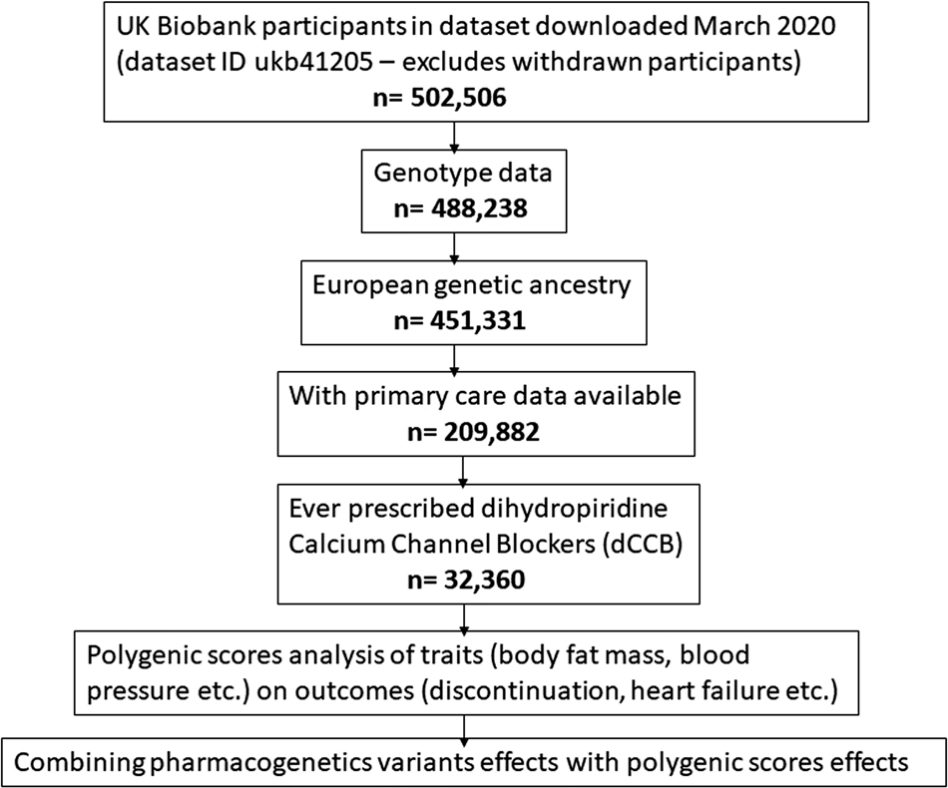
Flowchart of the methodology. The study has two steps: 1) Testing the associations between polygenic scores of patient characteristics and dCCB related adverse events, 2)  Combining the effects with polygenic scores to identify patients at most risk.

Primary analysis included 451,367 participants (93% of 481,000 with genotype data available [4, 22]) identified as European (determined by genetic clustering, as explained previously [23]). After subsetting to those with dCCB prescribing data (see Fig. 1 flowchart) the sample sizes from other genetic backgrounds were too small to study.

### Polygenic score

For the following patient characteristics reported to alter dCCB pharmacokinetics [5–11], we calculated polygenic scores by summing the number of risk increasing alleles, multiplied by the published effect, using independent genome-wide significant (*p* < 5*10–8) variants reported by publicly available large-scale genome wide association studies (GWAS) in European-like people from the Open GWAS platform [24] (see Supplementary Methods for more details on polygenic score derivation and software and Supplementary Table 1 for the genetic variants used in each polygenic score). We also derived a polygenic score for heart failure based on a recent GWAS [25]. We were not able to generate a polygenic score for ‘discontinuation of dCCB prescribing’ because there is not available GWAS for this outcome (see next section for rationale for the outcomes). See Table 1 for the traits and GWASs used in the polygenic scores.

**Table 1:** List of traits and GWASs used in polygenic scores.

Trait	Open GWASID or paper Reference	UKB only	Number of variants*
SBP	Evangelou 2018 [50]		240
DBP	Evangelou 2018 [50]		297
Body fat mass	UKB-b-19393 (IEU open gwas)	Yes	403
Appendicular lean mass	ebi-a-GCST90000025 (IEU open gwas)	Yes	630
Wait to hip ratio	Pulit 2019 [33]		266
eGFR ^	Pattaro 2015 [28]		49
Urinary Sodium	Zanetti 2019 [51]		37
Serum Calcium	O’Seaghdha 2013 [35]		7
Lipoprotein A	Burgess 2018 [36]		36
Heart failure	Shah 2020 [25]		12

See Supplementary Methods and Supplemetary Table 1 for details on variant selection and polygenic score creation. *Number of single nucleotide polymorphisms showed significant associations with the studied trait in the genome wide association analysis. ^ We coded higher eGFR polygenic score to correspond with worse kidney function (as presented by Pattaro 2015 [28]).

### Primary analysis: survival analysis

We aimed to extend our previous pharmacogenetics study [4] in 32,000 UKB dCCB patients where we tested associations between 23 genetic variants and dCCB adverse events using primary care and secondary care data, adding the effects of polygenic scores of patients’ characteristics on the same outcomes studied (see Fig. 1 for the methodology flowchart). In our pharmacogenetic analyses [4], the significant results were for: *NUMA1* rs10898815 A allele, increasing the risk of treatment switch (significant after Benjamini–Hochberg adjustment for multiple statistical testing: adjusted *p* = 0.04); and *RYR3* rs877087 T allele, increasing the risk of heart failure. For both the variants the prior evidence available on these genetic variants increased the plausibility of the associations observed [26, 27].

We used Cox proportional hazards regression models adjusted for sex, age at first prescription and genetic principal components 1 to 10 (Data-field 22009), to account for population substructure. We chose outcomes affected by pharmacogenetic variants in our previous study [4]. The primary outcomes were incident heart failure (HF) diagnoses and discontinuation of dCCB prescribing. We opted to use discontinuation over ‘switching antihypertensive treatment’ to better capture patients who are no longer prescribed dCCBs for any reason. Secondary outcomes were incident coronary heart disease (CHD: myocardial infarction or angina) and chronic kidney disease (CKD). Patients were included in the analyses if they had at least 2 dCCB prescriptions in a year and were older than age 40 at the first prescription (details described previously [4]). Patients entered the model on the date of first prescription and exited on the date of event, or were censored (date described in the discontinuation model).

We first conducted analyses with continuous polygenic scores, and then repeated the analyses with the 3 tertiles of polygenic scores to provide interpretable estimates of relative risk between groups without relying on small numbers of participants with extreme values. For the models using continuous polygenic scores, we standardized the scores (to give mean=0 and standard deviation = 1) to allow comparison of effect sizes between the different scores and outcomes. We used a Benjamini-Hochberg multiple testing correction.

STATA (v15.1) software and R (v4.2.1) were used for the analyses. ‘stset’, ‘stcox’ commands in STATA, and ‘coxph’ from the ‘survival’ package (v3.4-0) in R was used to fit Cox proportional hazards models.

### Combining pharmacogenetics and polygenic scores

We categorized patients to identify who is at most risk for HF and discontinuation risks based on the presence of high polygenic score and/or pharmacogenetic variant, and conducted survival analysis as described above. The “high-risk” polygenic score group includes patients in the top third of genetic liability for at least one polygenic score, and none of the first-tertile scores; the “low-risk” score group includes patients with at least one of the bottom third scores, and none of the third-tertile scores. We also tested the interaction effect between genotypes and polygenic scores.

Carrying at least one rs877087 T allele in *RYR3* (prevalence of 46% in UKB) increased the risk of HF with the HR 1.13 (95% CI 1.02–1.25) compared to non-carriers in UKB dCCB patients previously [4]. We compared patients in different risk groups based on the presence of T allele and high polygenic scores for body fat mass, lean mass, and lipoprotein A scores as these increased the risks for HF (see Results). We did not include the heart failure polygenic score to focus on patient characteristics that might be available in clinical practice.

Patients with rs10898815 GA (HR = 1.10, 95% CI 1.01 to 1.21) and AA in *NUMA1* (HR = 1.18, 95% CI 1.07 to 1.31), and rs776746 TT in *CYP3A5* (HR 1.87, 95% CI 1.26 to 2.78) were at increased risk of discontinuing dCCBs compared to their common homozygotes. We only took rs10898815 into account here due to the low prevalence of rs776746 TT (0.5% in this cohort). The same comparison model is used here as above, examining for body fat mass, and A allele of rs10898815.

We observed dominant effects for rs877087 T allele and rs10898815 A allele, so we modeled the carriers (heterozygotes plus homozygotes) compared to non-carriers (homozygous reference) throughout.

### Sensitivity analyses

We (1) repeated the combining model adjusting for additional antihypertensives during dCCB prescription, (2) used Cox model for discontinuation and rs10898815 in other antihypertensive, (3) used summary data Mendelian randomization methods (as opposed to “one sample” methods employed in the main analysis) to access a larger group of roboust estimators for checking the MR assumptions, (4) excluded existing HF diagnoses in the analysis of polygenic scores on HF risk. See Supplementary Information for details.

## Results

There were 32,360 (45.6% female) participants with primary care records meeting the inclusion criteria. The mean age at first dCCB prescription was 61.3 (SD 7.7) years and the median number of prescriptions per year was 8.2 (interquartile range [IQR] 6.6 to 13, range 2 to 25). The mean prescribing period was 5.9 (standard deviation [SD] 5.2) years, and the median was 4.4 (IQR 1.6 to 9.1). See Table 2 for patient characteristics.

**Table 2:** Characteristics of UKB patients prescribed dCCB.

GP prescribed	
Number of participants	32,360
Females, *n* (%)	17,590 (45,6)
Age at first prescription	
Minimum: maximum	40:79.3
Mean (SD)	61.3 (7.7)
Number of prescriptions in a year	
Minimum: maximum	2:25
Mean (SD)	9.6 (4.4)
Years between first and last prescription	
Minimum: maximum	0.08:39.9
Mean (SD)	5.9 (5.2)
MI/angina^^ pre prescription of dCCBs	3026 (7.7)
CKD^^ pre prescriptions of dCCBs	229 (0.6)
Heart failure^^ pre prescriptions of dCCBs	333 (0.8)
Prescribed other antihypertensive during Dccb	23,971 (61)
Discontinue dCCBs	10,095 (31.4)
MI/angina^^ post prescription of dCCBs	7430 (18.9)
CKD^^ post prescriptions of dCCBs	2940 (7.5)
Heart failure^^ post prescriptions of dCCBs	2292 (5.8)

European-ancestry participants with > 1 dCCB prescription in the available GP prescribing data.

*CKD* chronic kidney disease, *dCCB* dihydropyridine calcium-channel blocker.

^^Hospital diagnosed diseased.

### Polygenic scores

**Fig. 2:**
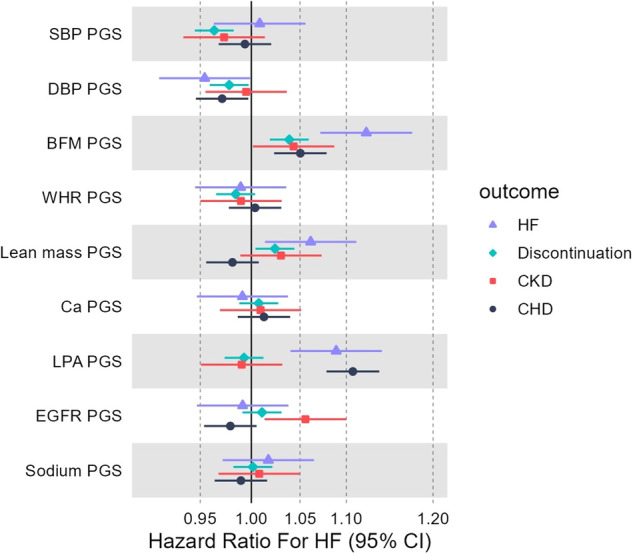
Forest plot of associations between patients’ characteristics polygenic scores and the risks of increased dCCB adverse events and discontinuation in 32,000 UK Biobank patients. SBP Systolic blood pressure, DBP Diastolic blood pressure, BFM Body fat mass, WHR Waist-to-hip ratio, Ca Serum Calcium^+2^, LPA Lipoprotein A, EGFR Estimated glomerular filtration rate, HF Heart failure, CKD Chronic kidney disease, CHD Coronary heart disease. HF, CKD, and CHD are diagnoses of hospital records. Time to event analyses between listed polygenic scores and hospital diagnosed heart failure, chronic kidney disease, coronary heart disease, and dCCB discontinuation in European-like aged 40+ years patients with at least two dCCB prescription. The models are adjusted for sex, age at first dCCB prescription and principal genetic components.

Of the nine polygenic scores tested, four were positively associated with increased risk of one or more studied dCCB adverse events (Fig. 2): body fat mass, lean mass, lipoprotein A, and eGFR. Two were negatively associated: SBP and DBP. See corrected *p*-values in Supplementary Table 2.

**Body fat mass polygenic score** was positively associated with increased risk of dCCB discontinuation (Hazard Ratio [HR] per SD increase in polygenic score 1.04: 95% Confidence Intervals [CI] 1.02–1.06, *p* = 1.3 × 10^−4^), heart failure (HF) and coronary heart disease (CHD) in Cox proportional-hazards models (HR_HF_ 1.12: 95% CI 1.07–1.18, *p* = 9.6 × 10^−7^; HR_CHD_ 1.05: 95% CI 1.02–1.08, *p* = 2.5 × 10^−4^). For details see Supplementary Table 2. To illustrate the effect another way, we stratified the participants into tertiles (three equally sized groups) of polygenic score. Those in the highest body fat mass polygenic score tertile had 31% increased risk of HF (HR = 1.31, 95% CI 1.17 to 1.47, *p* = 4 × 10^−6^), 10% increased likelihood of discontinuation (95% CIs 1.05 to 1.15, *p* = 1 × 10^−4^), and 13% increased risk of CHD (95% CI 1.06–1.21, *p* = 10^−4^), compared to the lowest third (Supplementary Table 3). The mean difference in measured body fat at UK Biobank baseline assessment between the top and bottom tertiles was 2.2 kg (95% CI 2.09–2.36) in a linear regression model adjusted for age, sex and the top 10 genetic principal components.

**Lean mass polygenic score** was associated with increased risk of HF (HR per SD increase in polygenic score 1.06: 95% CI 1.01–1.11, *p* = 0.01) and discontinuation (HR 1.02, 95% CI 1–1.04, *p* = 0.02, *p* = 0.06 after multiple testing correction). Those in the highest third of lean mass polygenic score had increased risks of HF (HR 1.14, 95% CI 1.02 to 1.27, *p* = 0.02) compared to the bottom third.

**Lipoprotein A polygenic score** was associated with increased risk for HF and CHD (HR_HF_ 1.09: 95% CI 1.04–1.14, *p* = 2.7 × 10^−4^ and HR_CHD_ 1.11: 95% CI 1.08–1.14, *p* = 4.5 × 10^−14^). Those in the top third had increased risk of HF (HR 1.20, 95% CI 1.07–1.34, *p* = 10^−3^) and CHD (HR 1.27, 95% CI 1.19–1.35*, p* = 3 x 10^−14^) compared to the lowest third despite the dCCB treatment.

**Systolic and diastolic BP scores** were associated with lower discontinuation rates (HR_SBP_ per SD increase in polygenic score 0.96, 95% CI 0.94-0.98 and HR_DBP_ per SD increase in polygenic score 0.98, 95% CI 0.96–0.99) (Fig. 2 and Supplementary Table 2). Patients at the highest third of SBP or DBP polygenic score had lower discontinuation rates, HR _SBP=_ 0.92 (95% CI 0.88–0.97) and HR _DBP_ = 0.95 (95% CI 0.90–0.99) compared to the lowest third.

**eGFR polygenic score** (where higher values corresponds with lower measured eGFR, as presented by Pattaro 2015 [28]) increased risk of chronic kidney disease (CKD) during dCCB treatment, with the highest third having 1.12 (95% CI 1.01–1.24, *p* = 0.03) times the risk versus the lowest (Supplementary Table 3).

**Heart failure (HF) polygenic score** was associated with increased risk for HF despite the dCCB treatment (HR per SD increase in polygenic score 1.14, 95% CI 1.09–1.19, *p* = 9.7 × 10^−9^). Patients at the highest third of HF polygenic score had 34% of higher risk for HF (HR = 1.34, 95% CI 1.20 to 1.50, *p* = 4 × 10^−7^) versus the bottom (Supplementary Table 3).

### Pharmacogenetics and polygenic scores

Pharmacogenetic variants were independent predictors of adverse outcomes in combined analysis with the above polygenic scores:

#### Heart failure

**Fig. 3:**
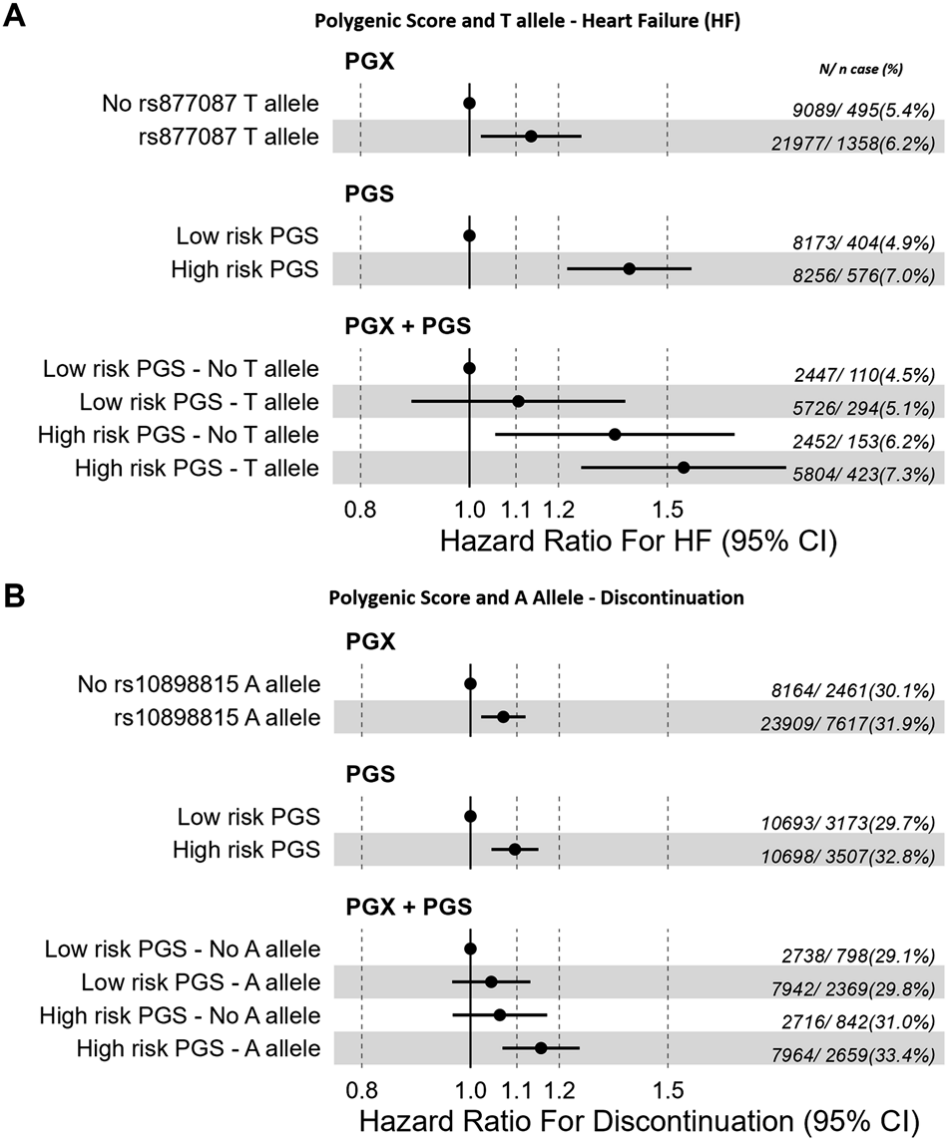
Time to event analysis between pharmacogenetic variant alone (A), polygenic score alone (B) and both together (C), with adverse events. Time to event analyses between outcomes and: **A** PGX: pharmacogenetic variants only (rs877087 T for heart failure and rs10898815 A for discontinuation) versus no variant; **B** PGS: polygenic score high risk only (either or all top third and not any bottom third; body fat mass+ lean mass+ lipoprotein A for heart failure and body fat mass for discontinuation) versus low risk score (either or all bottom third and not any top third); and **C** PGX + PGS: polygenic score and pharmacogenetic variant together. We analyzed European-like aged 40+ years patients with at least two dCCB prescription. The models are adjusted for sex, age at first dCCB prescription and principal genetic components.

Male patients prescribed dCCB were 1.8 times more likely to develop HF compared to female patients prescribed dCCB adjusting for age at first prescription, 10 genetic principal components and assessment center (HR = 1.8, 95% CI 1.6 to 2). In a Cox proportional hazards regression model for incident HF after initiating dCCB treatment, rs877087 T allele and polygenic scores for body fat mass, lean mass, and lipoprotein A had significant, independent effects (HR_rs877087_ 1.13, *p* = 0.02; HR_body fat mass_ 1.08, *p* = 1.7 × 10–6; HR_lean mass_ 1.04, *p* = 0.02, HR_Lipoprotein A_ 1.06, *p* = 2 × 10–4), after adjusting for age at treatment initiation, sex and the top 10 genetic principal components. T allele alone versus no T allele increased HF risk with a HR of 1.13 (95% CI 1.02–1.26, *P* = 0.02) and high polygenic score alone had HR of 1.39 (95% CI 1.22–1.58, *p* = 4.6 × 10^−7^) versus low scores adjusted for covariates. Estimates were larger with the presence of high polygenic scores and pharmacogenetic risk allele together with no significant interaction (*p* = 0.8): the high risk PGS-T allele (7.3% prevalence of HF) HR 1.55 (95% CI 1.26–1.91, *p* = 4.2 × 10^−5^) versus low risk PGS-No T allele (4.5%); High risk PGS-No T allele HR 1.35 (95% CI 1.05–1.72, *p* = 0.02); Low risk PGS-T allele HR 1.11 (95% CI 0.89–1.38, *p* = 0.37) (Fig. 3, Supplementary Table 4).

#### Discontinuation

rs10898815 A allele carrier status and polygenic scores for body fat mass also had significant, independent effects (HR_rs10898815_ 1.07, *p* = 0.002; HR_body fat mass_ 1.03, *p* = 10–4). High risk PGS-A allele together had larger effect on discontinuation risk with no significant interaction terms (*p* = 0.7): HR 1.16 (95% CI 1.07–1.25, *p* = 3.3 × 10^−4^).

See Supplementary Results for the sensitivity analyses.

## Discussion

We aimed to estimate the combined effect of polygenic scores with pharmacogenetic variants on dCCB adverse outcomes using a large-scale study of 32,000 community patients prescribed dCCB. Previous genetic research in hypertension has modeled pharmacogenetic variants [29] or polygenic scores [30], but not together. We previously reported that variants in *RYR3* and *NUMA1* [4] increased risks for heart failure (HF) and treatment discontinuation respectively in the same patient sample, although effect sizes were modest. Here we studied polygenic scores for individual patient characteristics reported to alter dCCB pharmacokinetics. By analysing genetically predicted traits we aimed to minimize confounding, as genetic variants are inherited at conception and are unaffected by later exposures or downstream effects of disease. We found that genetically predicted body fat mass, lean mass, and lipoprotein A were associated with adverse clinical outcomes in hypertensive patients treated with dCCBs. Additionally, genetic liability to HF increased incident HF risks in patients prescribed dCCBs. Though overall estimates for pharmacogenetic variants are modest, risk of adverse outcomes are considerably higher in combination with high polygenic scores; suggesting genetic information could have utility in identifying the subset of hypertensive patients with substantially raised risks of adverse events whilst on CCB treatment.

Adiposity is a common risk factor for many diseases, specifically with cardiovascular diseases [31–33], with genetics supporting the well-established causal role of adiposity and cardio-metabolic risk [31]. We extend the literature by exploring associations in pharmacogenetics. We found that patients with higher genetically predicted body fat mass were more likely to have HF, CHD, CKD, and to discontinue treatment, compared to those with low genetic burden, despite the dCCB treatment. The pathway between higher body fat mass and clinical outcomes is complex, likely two-fold: (1) via non-pharmacogenetic effects on cardio-metabolic pathways, and (2) dCCBs are highly lipophilic, therefore greater body fat mass reduces medication effectiveness. Patients with higher fat mass may need larger doses [5, 6].

The causal role of lipoprotein A on atherosclerotic cardiovascular diseases is supported by several Mendelian Randomization studies [34–36]. Although dCCBs were reported as being protective for experimental atherosclerosis [37, 38], our results show that genetically predicted lipoprotein A increased risk of HF (HR 1.09: 95% CI 1.04–1.14) and CHD (HR 1.11: 95% CI 1.08–1.14) in dCCB patients. We were unable to test the associations with direct measured lipoprotein A at treatment initiation, however others have demonstrated similar predictions on cardiovascular diseases between the polygenic score and a direct measurement [39].

A randomized controlled trial (RCT) [8] found that age and baseline BP affect dCCB response in ~60 patients. In UK Biobank, a study found that in patients reporting antihypertensive use at baseline genetically predicted SBP was associated with uncontrolled BP (OR 1.70; 95% CI: 1.6–1.8, top vs. bottom quintile) [40]. Another UK Biobank study found that high SBP polygenic score at baseline increased cardiovascular risk 1.04-fold in untreated hypertension and 1.06-fold in treated hypertension [41]. In our study, high SBP and DBP polygenic scores are associated with decreased the risk of discontinuation, and high DBP polygenic score decreased risk of HF and CHD. We hypothesize that those with the most severely increased blood pressures tend to continue treatment compared to those with more moderately increased pressures (although we lack data to test this directly). It might also suggest that patients with higher predisposition to hypertension may get better overall benefit from the dCCB treatment, similar to the finding of two RCT on lipid-lowering showing that patients with highest genetic risk of atherosclerosis had better benefit from lipid-lowering treatment [42].

Serum calcium and urinary sodium were negatively associated with BP response in ~52 Finnish men in another 4-week RCT [10], however we found no associations with serum calcium and sodium polygenic scores between outcomes in 32,000 patients. Calcium channel blockers are excreted renally, so we tested whether genetically predicted eGFR affects dCCB response: high eGFR polygenic score increased the risk of CKD in dCCB patients.

dCCBs are accepted as safe options for the first line treatment of hypertension in non-black patients aged 55 and over, and reported to have protective effects in HF by the UK National Institute for Health and Care Excellence [43]. However, our results suggest that specific patients at higher genetic risk for HF (i.e., those with higher genetic liability to HF, plus higher genetically predicted body fat mass, lean mass, and lipoprotein A, and carrying the pharmacogenetic *RYR3* variant rs877087 T-allele) have worse outcomes compared to those with lower genetic risk. Individual-level predictions incorporating this information might improve clinical outcomes in hypertension treatment.

Including genetic and non-genetic risk factors that could alter estimation of treatment response in routine prescribing and improve outcomes. Under the evidence-based medicine regimes, more robust studies are required to implement new prescribing methods. In this study, using polygenic scores we minimize the effect of unmeasured confounders as genetic variants are fixed at conception and reflect lifetime exposure to the risk factor [44], with each variant essentially inherited at random to other variants and health risks, thus providing a natural experiment comparable to a randomized clinical trial. The potential clinical utility of polygenic scores and pharmacogenetics is not yet realized [45, 46]. We believe this is the first study examining polygenic scores in combination with pharmacogenetic variants in a large cohort of 32,000 community patients prescribed dCCBs over 5.9 years, using the primary care linked data reflecting the routine clinic. Studies such as these will inform efforts in personalized medicine. Simulation results from a UK Biobank study navigating the effect of winner’s curse and weak instrument bias in the case of sample overlap [47] shows that bias is minimal when only strong instruments are used. Thus any bias in our analysis is thought to be minimal considering our strongly instrumented body fat, despite using UK Biobank in both the construction of the PRS and the main analysis. However, for increased robustness, future studies should replicate the associations for polygenic scores ascertained from UK Biobank (fully: BFM, LM and partly: SBP and DBP) in different cohorts.

Study limitations include not testing actual measurements of risk factors (routine GP data does not systematically measure these at treatment initiation), nor analyzing BP response due to sparsity of data available (the majority of patients were missing GP-measured BP within 2 months of treatment initiation). We were not able to analyze CCB ‘dose’ due to missing/inconsistent recording of data, and high intra-patient variability. Further work is needed before specific treatment recommendations could be made for the high-risk group (e.g., dose adjustments, or alternative treatment prescribed). Future studies are planned to extend the data analysis to incorporate untreated individuals and extend analytical methods – such as our recently published TWIST framework [48]. Further studies are needed to construct genetic scores for discontinuation in dCCB patients, and extend the approach reported, including data on populations of non-European ancestry, and analyzing a wider range of alleles Replication of our results and examination of drug-drug-gene interactions are needed.

In summary, clinical outcomes seen in patients prescribed the common antihypertensives dihydropiridine calcium channel blockers were better estimated when incorporating polygenic risk scores related to adiposity and lipoprotein A together with pharmacogenetic variants. Hence, efforts to personalize treatment regimes should consider multiple genetic risk factors to improve patient outcomes. Combining pharmacogenetic and polygenic score data may have wider applications for prescription optimization of other medications, especially as genome wide genotype data becomes more widely available in routine clinical practice.

### Supplementary information

Supplementary Information

Supplementary Tables

## Data Availability

The genetic and phenotypic UK Biobank data are available upon application to the UK Biobank (www.ukbiobank.ac.uk/register-apply). The derived data fields used in our analysis will be available via the UK Biobank, search for application number 14631. We are not able to share these directly.
