# Supplementary material for: Polygenic scores for cardiovascular risk factors improve estimation of clinical outcomes in CCB treatment compared to pharmacogenetic variants alone
Source: Pharmacogenomics J. 2024 Apr 17;24(3):12. doi: 10.1038/s41397-024-00333-2 (PMC11023935; doi:10.1038/s41397-024-00333-2)
Supplement: Supplementary file 1 — Supplementary Information [file 41397_2024_333_MOESM1_ESM.docx]

**Effect of pharmacogenetic variants and polygenic scores of patient characteristics combined on adverse outcomes during Calcium Channel Blocker treatment**

Türkmen et al.

**Supplementary Information**

[Supplementary Methods 1](#_Toc149828648)

[List of antihypertensive medications identified, including brand names 1](#_Toc149828649)

[Disease ascertainment 1](#_Toc149828650)

[Genotyping of UK Biobank 1](#_Toc149828651)

[Polygenic scores 1](#_Toc149828652)

[Associations between genotypes and adverse events 2](#_Toc149828653)

[Sensitivity analyses 2](#_Toc149828654)

[1) Two-sample MR methods 2](#_Toc149828655)

[2) Excluding existing heart failure diagnoses 3](#_Toc149828656)

[Supplementary Results 4](#_Toc149828657)

[1) TwoSampleMR 4](#_Toc149828658)

[IVW MR Scatter Plots 4](#_Toc149828659)

[2) Excluding existing heart failure 6](#_Toc149828660)

[References 7](#_Toc149828661)

# Supplementary Methods

## List of antihypertensive medications identified, including brand names

**Amlodipine**: Norvasc®, Katerzia®, Istin®, Amlostin®, Astudal®, Amlodipine®; **felodipine**: Plendil®, Cardioplen®, Felogen®, Felendil®, Cabren®, Felotens®, Preslow®, Keloc®, Vascalpha®, Neofel®, Parmid®; lacidipine: Lacipil®, Motens®, Caldine®, Molap®, Lacidipine®; nisoldipine: Sular®, **lercanidipine**: Zanidip®, Lercanidipine®, **nimodipine**: Nimotop®, Nymalize®, **nifedipine**: Adalat®, Afeditab®, Procardia®, Adipine®, Nifedipress®, Tensipine®, Dexipress®, Valni®, Adanif®, Neozipine®, Nidef®, Nifedipine®, Fortipine®, Coracten® ; **nitrendipine**: Baypress®, **nicardipine**: Cardene®, Nicardipine®.

## Disease ascertainment

Primary and secondary care health records were used to examine the dCCB related adverse events.

Cardiovascular events from hospital admissions records were available up to 14 years follow-up after baseline assessment (HES in England up to 30 September 2020: data from Scotland and Wales censored to 31 August 2020 and 28 Feb 2018, respectively), covering the entire period up to the date of censoring of primary care prescribing data. Diagnosis of MI/angina was ascertained using ICD-10 codes I20*, I21*, I22*, I23*, I24* and I25*; ischemic stroke was using ICD-10 codes I63*; chronic kidney disease (CKD) using ICD10 codes N18* and Y84.1; and heart failure using ICD10 codes I50* and J81*.

## Genotyping of UK Biobank

Directly genotyped genetic variants (n=805,426) were ascertained in 488,377 UK Biobank participants, which used two almost identical platforms sharing >95% of variants: the Affymetrix Axiom UKB array (in 438,427 participants) and the Affymetrix UKBiLEVE array (in 49,950 participants). Extensive quality control was applied by the central UK Biobank team (1). Genotype imputation increased the number of genetic variants to ~96 million in 487,442 participants (1).

## Polygenic scores

To construct the polygenic scores used in the manuscript we used the following approach: 1) identify the independent lead variants (p<5*10^-8^) reported in the GWAS paper (or by the IEU Open GWAS platform for “whole body fat mass” and “appendicular lean mass”); 2) check the UK Biobank imputed (version 3) data whether the RSID was available with matching alleles, imputation quality >0.3, minor allele frequency>0.001, Hardy-Weinberg p-value>5*10^-8^, and missing proportion<5*10^-8^; and 3) derive the polygenic score based on the number of trait-raising alleles, weighted by the published effect (see Supplementary Table 1 for details on included variants). In mathematical notation the polygenic score variable $\hat{S}$ is derived as:

$$\hat{S}= \sum_{n=1}^{N} X_{n}\hat{\beta}_{n}$$

where $X_{n}$ is the number of trait-increasing alleles of variant $n$ weighted by their published effect size estimate $\hat{\beta}_{n}$, for each of the $N$ genetic variants $(n=1\ldots N)$.

We used Plink v1.9 with option ` --score` to compute the polygenic scores for each trait using the provided list of variants weighted by their published effect size.

## Associations between genotypes and adverse events

These (hospital-diagnosed CHD (MI/angina), HF, and CKD) were estimated using Cox proportional hazards regression models. They were adjusted for age at first prescription, sex, and genetic principal components 1 to 10 (to adjust for population genetic ancestry) in patients who were prescribed dCCBs by their GPs (General Practice). We included patients if they have had at least two prescriptions and more than 2 months of dCCBs treatment. Patients exited the model on the hospital inpatient record censoring dates i.e., for cardiovascular and renal outcomes we therefore performed an ‘intention to treat’ analysis, reducing any bias due to discontinuation before diagnosis of adverse outcomes (e.g., discontinuing dCCB treatment and having heart diseases as a result).

For the analysis of CKD diagnosis during dCCB prescribing we first performed the analysis in all patients, then repeated as secondary analysis excluding patients with CKD diagnosis prior to first dCCB prescription. For the analysis of acute MI and angina we did not exclude prevalent cases as worsening angina and acute MI are reported as a caution for patients with coronary arteria disease (2) in the prescribing information, and are therefore indications for dCCB prescribing.

## Sensitivity analyses

In our previous study where we examined the pharmacogenetic variants possibly affecting dCCB response in UK Biobank, we conducted sensitivity analyses using Cox proportional hazards regression models between pharmacogenetic variants and outcomes; assessed for additional antihypertensives within the dCCB prescription time, analysis of amlodipine only and other dCCBs separately, and analysis of unrelated participants only (3). The results of the analyses remained consistent with the main analysis; therefore, we did not see the need to repeat all the sensitivity analysis results here. We repeated the first one for possible drug-drug-gene interaction in this study

### 1)Additional antihypertensives

To attempt to assess to drug-drug-gene interaction, we repeated the combined model (pharmacogenetic variant + polygenic score) adjusting for additional antihypertensive medications prescribed during dCCB treatment (n=23,971, 61%) (See (3) for the details of the variable).

**2) *NUMA1* variant rs10898815 and discontinuation in other antihypertensives**

Previous studies showed associations between rs10898815 and CCB treatment response(3,4). However, to reassure the drug specific effect, we conducted a sensitivity analysis for discontinuation in some other antihypertensives. We used Cox proportional hazards regression models adjusting for sex, age at first prescription, and genetic principal components 1 to 10 (to adjust for population genetic ancestry) in patients who were prescribed Angiotensin-Converting Inhibitors (ACEIs), Beta Blockers (BB) separately. The method was same with the primary discontinuation analysis. See medications in (3).

###

### 3) Two-sample MR methods

a) To test whether associations between genetically predicted body fat mass and dCCB clinical outcomes (HF, CHD and discontinuation) – the primary results – were unbiased (no unbalanced pleiotropy) and causal; it would be more effective to conduct future randomized controlled trials specifically designed to test these associations in patients where the causal relationships are established.

We used summary statistics (or “two sample”) MR methods, to exploit the advantages of the different approaches. We used R package `TwoSampleMR` (v0.5.6) to estimate the inverse-variance weighted (IVW) effect of each individual exposure-associated variant on the outcome – akin to the estimate from the polygenic scores. The IVW (and polygenic score approach) assumes there is balanced horizontal pleiotropy (i.e. with a zero mean); we therefore additionally applied ‘weighted median’ (assumes less than 50% of the weight in the analysis comes from invalid instruments) and ‘MR-Egger’ (allows for unbalanced pleiotropy providing genetic variants’ effect on body fat mass is not correlated with their pleiotropic effects on the outcome) approaches to test robustness of the estimate. The MR-Egger method additionally estimates an intercept and deviation from the null is used to test for possible unbalanced pleiotropy. We also calculated the $I_{GX}^{2}$ statistic to check for bias due to the `NO Measurement Error’ (NOME) assumption (i.e., where the SNP-exposure association is assumed to be known, rather than estimated). Finally, ‘leave one out’ analysis tests whether the result is overly influenced by a single variant.

b) We have performed sensitivity analyses to assess the impact of sample overlap (5) (the chief concerns are bias due to Winner’s Curse and also Weak Instrument Bias). We used the Two-Sample MR methods for Body Fat Mass to investigate the impact:

First, for Winner’s curse, we followed the approach in (6) using R syntax from (7) to correct instrument betas and standard errors. Although this correction helps to minimise bias, it does not fully account for sample overlap.

Second, we investigated the effect of weak instrument bias by repeating the above analysis excluding instruments with body-fat-mass-p>1x10^-9^.

### 4) Excluding existing heart failure diagnoses

NICE report that amlodipine, nifedipine, felodipine and nicardipine do not cause deterioration in heart failure(8). This advice could mean that more severe patients might have been prescribed dCCBs. In order to minimize the potential for selection bias, we repeated the model for heart failure excluding patients with existing heart failure diagnoses prior to the dCCB prescriptions.

# Supplementary Results

**Sensitivity Analyses**

## Adjusting for additional antihypertensives

Estimates were larger with the presence of high polygenic scores and pharmacogenetic risk allele together with no significant interaction (p=0.8): the high risk PGS-T allele prevalence of HF) HR 1.55 (95%CI 1.26-1.91, p=4.2x10^-5^) versus low risk PGS-No T allele, HR was 1.54, (95% CI 1.28-1.85, *p*=5.6x10^-6^ ) after adjustment for additional antihypertensives.

1. ***NUMA1* variant rs10898815 and discontinuation in other antihypertensives**

The effect of rs10898815 was not observed in other antihypertensive discontinuations. In patients prescribed ACEIs HR was 1.02, 95% CI 0.96-1.09, p=0.54; BBs: HR was 0.94, 95% CI 0.94-1.04, p=0.22).

## TwoSampleMR

1. In TwoSampleMR methods the weighted median and MR-Egger central estimates were consistent with those from IVW. In addition, we observed no significant evidence for bias due to unbalanced pleiotropy: MR-Egger intercept p=0.7 for heart failure, p=0.8 for discontinuation and p=0.2 for CHD (See Supplementary Table 5). The $I_{GX}^{2}$ statistic for body fat mass exposures = 0.98, indicating no violation of the NOME assumption, thus MR Egger estimates were valid.
2. All three results (without correction, with winner’s curse correction, and limited to stronger instruments) are highly consistent with each other, indicating that even if the samples were fully overlapped, the bias was minimal (see Supplementary table 5).

## IVW MR Scatter Plots

**Figure1-MR of body fat mass and heart failure in UK Biobank dCCB prescribed patients**


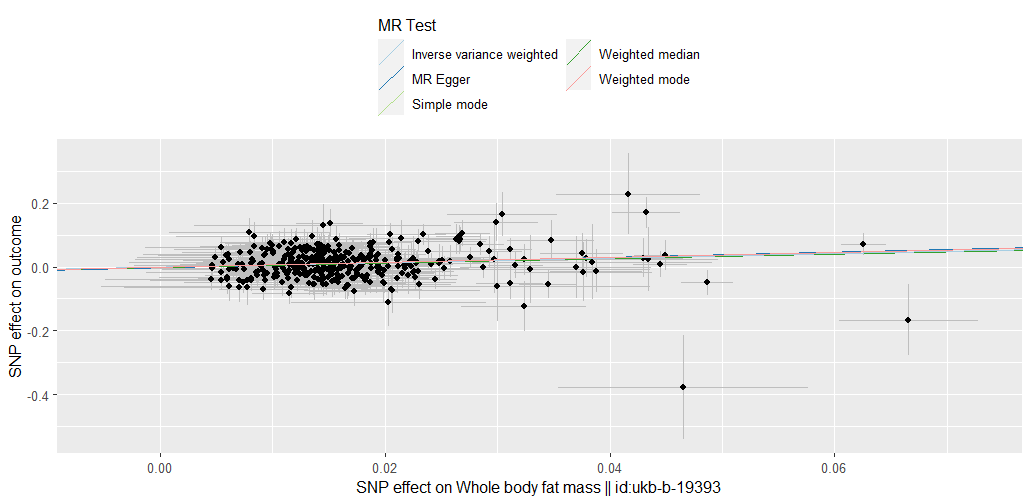


**Figure 2- MR of body fat mass and discontinuation in UK Biobank dCCB prescribed patients**


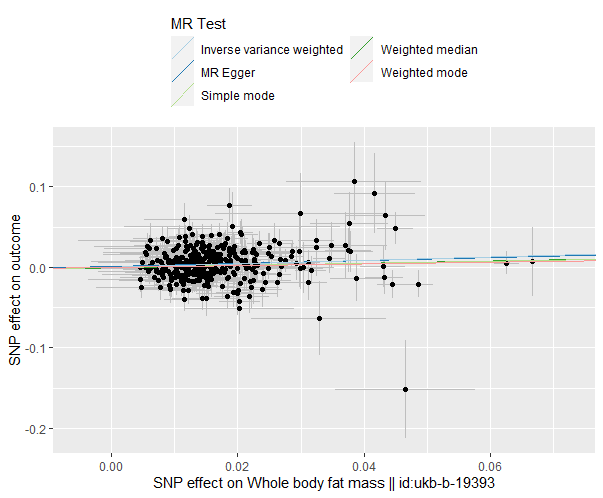


**Figure 3-MR of body fat mass and coronary heart disease in UK Biobank dCCB prescribed patients**


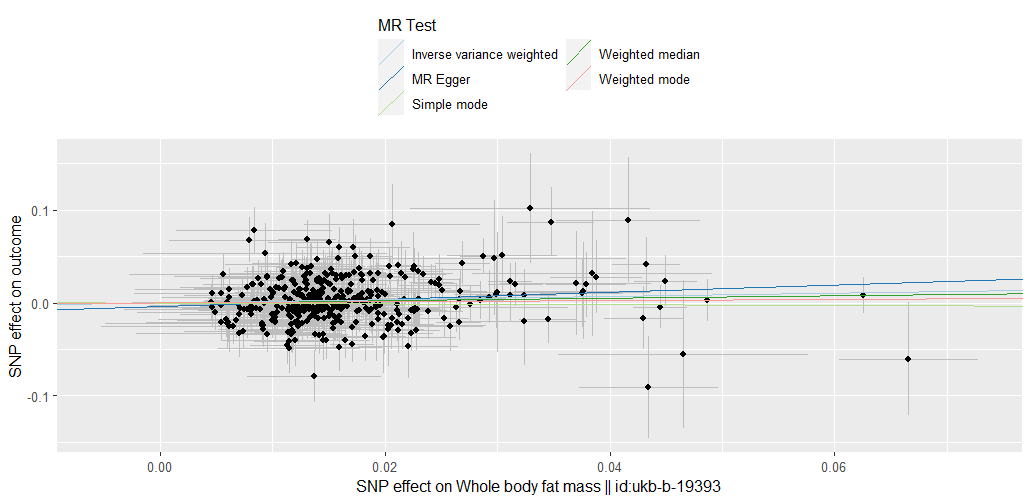


## 3) Excluding existing heart failure

After excluding existing heart failure diagnoses, 1,737 heart failure diagnoses remained (N total=30,819). The polygenic scores increasing the risk of heart failure remained significant with similar effect sizes (i.e. HR per SD increase in polygenic score of body fat mass 1.13, 95% CI 1.07-1.18, *p*=1.08*10**^-6^**, of lipoprotein A HR 1.09, 95% CI 1.04-1.14, p=3.6*10^-4^).

# References

1. Bycroft C et al. The UK Biobank resource with deep phenotyping and genomic data. Nature. 2018;562(7726):203–9.

2. Food and Drug Administration. NORVASC® (amlodipine besylate) Prescribing Information. 1987;1–12.

3. Türkmen D et al. Calcium‐channel blockers: Clinical outcome associations with reported pharmacogenetics variants in 32 000 patients. Br J Clin Pharmacol. 2022 Oct 6;bcp.15541.

4. Kamide K et al. Genome-wide response to antihypertensive medication using home blood pressure measurements: a pilot study nested within the HOMED-BP study. Pharmacogenomics. 2013 Nov;14(14):1709–21.

5. Bowden J et al. Unbiased estimation of odds ratios: combining genomewide association scans with replication studies. Genet Epidemiol. 2009 Jul;33(5):406–18.

6. Zhong H et al. Correcting ‘winner’s curse’ in odds ratios from genomewide association findings for major complex human diseases. Genet Epidemiol. 2010 Jan;34(1):78–91.

7. Liu R et al. Mid-life leukocyte telomere length and dementia risk: An observational and mendelian randomization study of 435,046 UK Biobank participants. Aging Cell. 2023;22(7):e13808.

8. Calcium-channel blockers | Treatment summaries | BNF content published by NICE [Internet]. [cited 2023 Jan 21]. Available from: https://bnf.nice.org.uk/treatment-summaries/calcium-channel-blockers/
